# *YABBY* Genes in the Development and Evolution of Land Plants

**DOI:** 10.3390/ijms22084139

**Published:** 2021-04-16

**Authors:** Marina A. Romanova, Anastasiia I. Maksimova, Katharina Pawlowski, Olga V. Voitsekhovskaja

**Affiliations:** 1 Department of Botany, St. Petersburg State University, Universitetskaya Nab. 7/9, 190034 Saint Petersburg, Russia; 2 Laboratory of Molecular and Ecological Physiology, Komarov Botanical Institute, Russian Academy of Sciences, ul. Professora Popova 2, 197376 Saint Petersburg, Russia; AEvkaikina@binran.ru; 3 Department of Ecology, Environment and Plant Sciences, Stockholm University, 106 91 Stockholm, Sweden; katharina.pawlowski@su.se; 4 Saint Petersburg Electrotechnical University “LETI”, ul. Professora Popova 5, 197022 Saint Petersburg, Russia

**Keywords:** abaxial domain, adaxial domain, ARP, telome theory, sterilization theory

## Abstract

Mounting evidence from genomic and transcriptomic studies suggests that most genetic networks regulating the morphogenesis of land plant sporophytes were co-opted and modified from those already present in streptophyte algae and gametophytes of bryophytes *sensu lato*. However, thus far, no candidate genes have been identified that could be responsible for “planation”, a conversion from a three-dimensional to a two-dimensional growth pattern. According to the telome theory, “planation” was required for the genesis of the leaf blade in the course of leaf evolution. The key transcription factors responsible for leaf blade development in angiosperms are YABBY proteins, which until recently were thought to be unique for seed plants. Yet, identification of a YABBY homologue in a green alga and the recent findings of YABBY homologues in lycophytes and hornworts suggest that YABBY proteins were already present in the last common ancestor of land plants. Thus, these transcriptional factors could have been involved in “planation”, which fosters our understanding of the origin of leaves. Here, we summarise the current data on functions of YABBY proteins in the vegetative and reproductive development of diverse angiosperms and gymnosperms as well as in the development of lycophytes. Furthermore, we discuss a putative role of YABBY proteins in the genesis of multicellular shoot apical meristems and in the evolution of leaves in early divergent terrestrial plants.

## 1. Introduction

**Figure 1:**
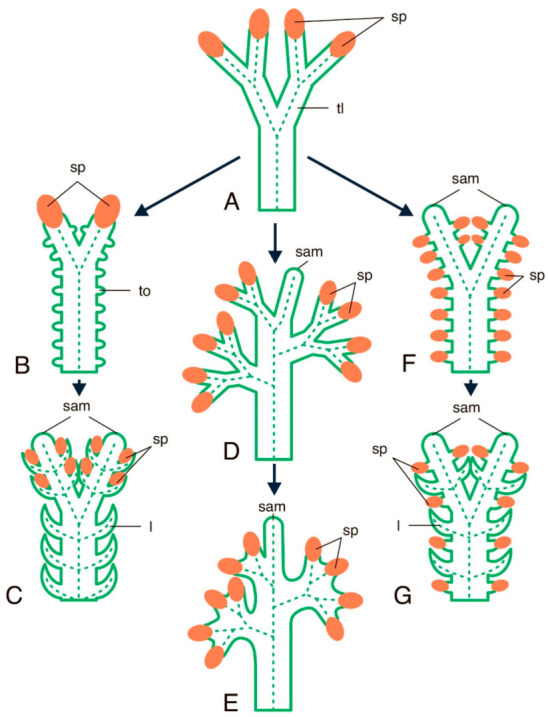
Summary of hypotheses on the origin of leaves in sporophytes of land plants. (**A**) Leaflesss rhiniophyte ancestor. (**B**,**C**) Successive stages of leaf origin as outgrowths of the cortex and epidermis of leafless telomes combined with the displacement of sporangia from terminal to lateral position. (**D**,**E**) Successive stages of leaf origin as a result of modification of the system of the leafless axes, or telomes (overtopping, planation and webbing). (**F**,**G**) Successive stages of leaf origin as a result of displacement of sporangia from a terminal to a lateral position combined with consequent sterilization of some sporangia. Schemes are based on descriptions and/or drawings of [1,2,3,4,5,8]. l—leaf, sam—shoot apical meristem, tl—telome, to—telome outgrowth, sp—sporangium. Dashed lines stand for the provascular and vascular tissues of stems and leaf traces.

Leaves are dorsoventral organs characterised in having determinate growth and being specialised for photosynthesis. The emergence of leaves, which occurred shortly after plants had conquered the land, became one of the most important events both in the evolution of plants and in the ecology of the Earth. However, the evolutionary origin of leaves is still under discussion [1,2,3,4,5,6,7,8,9,10]. Different viewpoints can be summarised into three scenarios based primarily on paleobotanical, morphological, and anatomical data: (1) the leaves of all plants share a common origin and are homologous to the system of the leafless axes, or telomes, of the first terrestrial plants (Rhyniophyta *sensu lato*), and their morphological differences in different taxa result from reduction or elaboration of these telome systems [3,4]; (2) while the leaves of most plants (called telome leaves, or megaphylls) indeed arose as a result of modification of the telome system, the leaves of some plants (at least of the lycophytes) that are termed enations or microphylls originated de novo as outgrowths of the cortex and epidermis of leafless telomes; therefore, there were two independent origins of plant leaves in evolution [1]; (3) leaves in different plant taxa originated independently more than two times through various mechanisms; the sterilisation of sporangia among them [2,5,6,8]. The three abovementioned hypotheses are summarized in Figure 1. Since the evolutionary origin of leaves represented the emergence of a new developmental programme or a modification of one (or more) already existing one(s), comparison of the genetic programmes regulating leaf initiation and leaf development in divergent taxa might help to shed light on the evolutionary origin of leaves.

For a number of flowering plants, it has been shown that leaf initiation in the peripheral zone (PZ) of the shoot apical meristem (SAM) results from the switch between indeterminacy and determinacy programmes. The above switch is controlled by the interaction between transcription factors (TFs) encoded by genes from *class I KNOTTED* (*KNOX*) family, which are markers of undifferentiated cells of the SAM, and four groups of “leaf development regulators”: (1) TFs encoded by the *ARP* genes (named after *ASYMMETRIC LEAVES1, ROUGH SHEATH2* and *PHANTASTICA*) [11], (2) small TFs that are unique for plants and encoded by members of the *YABBY* gene family [12,13], (3) members of the *KANADI (KAN)* gene family, wherein the members encode GARP-domain transcription factors [14], and (4) TFs encoded by representatives of class III of the *HD-ZIP* family [15]. Unlike the former three groups, HD-ZIPIII TFs do not act antagonistically but complementarily to the action of the class I KNOX TFs in the genesis of the SAM during embryogenesis and axillary bud initiation [16,17]. The transcriptional polarization of leaf primordia by leaf-specific TFs in the course of primordium initiation is a prerequisite for primordium development and emergence of a marginal meristem that produces the dorsoventral leaf blade in most dicot leaves. During leaf development, ARP and HD-ZIPIII TFs regulate the differentiation of the adaxial leaf domain (palisade parenchyma, upper epidermis and xylem), while KANADI TFs control the differentiation of spongy parenchyma, lower epidermis, phloem, and the abaxial domain [18,19], and YABBY TFs in combination with HD-ZIPIII and KANADI TFs regulate the outgrowth of the lamina [20,21].

Homologues of the *class I KNOX, KANADI,* and *HD-ZIPIII* genes were found in the genomes and transcriptomes of representatives of all land plant taxa; an *ARP* homologue was found in *Selaginella* spp. and its presence was implied in *Osmunda regalis* [7,8,22,23,24,25,26,27,28,29,30,31,32,33]. However, the hypotheses based on few studies that report the expression patterns of the above genes support different scenarios of leaf origin [7,25,26,28,34,35]. The presence of both KNOX and ARP homologues in the shoot tips of both lycophytes with microphylls and ferns with megaphylls has been interpreted as an indication of similar molecular regulation of organogenesis in these plant taxa and hence of a common evolutionary origin of leaves in all land plants [28]. However, it has to be taken into account that ARP was detected in the leptosporangiate fern *Osmunda regalis* using antibodies raised against maize ARP [28], and recent analyses show that the genomes of three other leptosporangiate ferns from the genera *Azolla, Salvinia,* and *Ceratopteris* do not contain an *ARP* gene [34]. Thus, the KNOX/ARP combination does not exist in all ferns, as it does not seem to exist in all lycophytes [36]. On the other hand, *KNOX* expression in the leaves of ferns and its absence in lycophyte leaves is interpreted as an indication of an independent origin of these two leaf types. It has been hypothesised that the absence of *KNOX* expression in lycophyte leaves does not result from its transcriptional repression, but reflects the absence of a potential for indeterminate growth of the *de novo* outgrowths, microphylls [25]. The colocalisation of the expression of *KNOX* and *ARP* in leaves of ferns, as opposed to the ARP-mediated *KNOX* repression in the primordia of angiosperm leaves, is considered as evidence that leaf genesis in ferns was independent of that in angiosperms [25]. Furthermore, the colocalisation of the expression of the above genes is also considered as an indication of shoot-like indeterminate growth of fern leaves [7,31]. Colocalisation of the expression of the *KNOX* and *ARP* homologues in the SAM of lycophytes and ferns led to the hypothesis that once these TFs jointly controlled dichotomous branching of the leafless axes in the first land plants, and then this developmental programme was subsequently modified and used for regulation of leaf origin [28]. Non-polar expression of *HD-ZIPIII* homologues and *KANADI* in lycophyte microphylls as opposed to their polar expression in megaphylls of both ferns and angiosperms is considered as an additional support of the scenario of an independent origin of these two leaf types [25,32]. On the other hand, expression of the *KANADI* homologues in the SAMs of ferns and the lack of their expression in SAMs of both lycopods and angiosperms [32] indicate that the evolution of the interaction between adaxial and abaxial determinants in non-angiosperm plants is far from resolved. At the same time, the expression of *HD-ZIPIII* and *KANADI* homologues in primordia of leaves and sporangia in both lycophytes and ferns led to the hypothesis that both microphylls and megaphylls originated in the course of modification of the sporangia developmental programme, and thus leaves of all land plants share deep homology [8,32].

Unlike *class I KNOX, ARP,* and *HD-ZIPIII*, the TFs encoded by *YABBY* genes have been regarded as unique to seed plants since no YABBY homologs could be identified in the genomes of *Physcomitrella patens*, *Marchantia polymorpha*, and *Selaginella moellendorffi* or in the transcriptomes of liverworts, bryophytes, lycophytes, or ferns [21,22,23,24,25,26,35]. This led to the conclusion that seed plant leaves were characterised by a unique laminar developmental programme absent from both microphylls and megaphylls of non-seed plants [21]. However, the recent identification of a *YABBY* homologue in the transcriptome of the homosporous lycophyte *Huperzia selago* [36] and its sister species *Huperzia serrata* [37], and later in the genomes of three hornwort strains of the genus *Anthoceros* [38] together with the finding of a YABBY-like protein in the green alga *Micromonas* [39] suggests that *YABBY* genes originated much earlier in land plant evolution than previously thought. Altogether, these data suggest that *YABBY* genes originated in the last common ancestor of land plants and later were lost in most lineages of seedless plants. This scenario might challenge the current concepts of the evolution of molecular programmes that led to the origin of leaves in land plants. Therefore, we set about to review the current data, available for diverse plant taxa, on phylogeny and putative functions of *YABBY* homologues and to speculate on their putative ancestral function and its evolutionary modifications.

## 2. Molecular Characterization of YABBY Proteins

**Figure 2:**
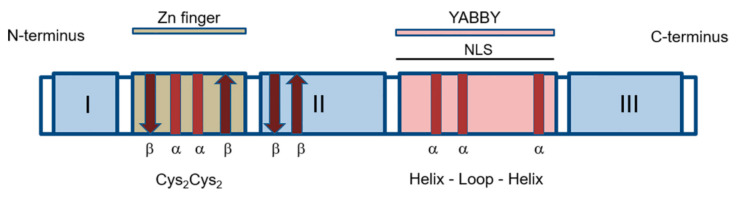
Structure of YABBY proteins. YABBY transcription factors are characterized by two highly conserved domains, the N-terminal Cys2/Cys2 zinc-finger and the C-terminal DNA-binding YABBY domain representing a helix-loop-helix motif with similarity to high mobility group domain [12]. Red rectangles labeled α and brown arrows labeled β represent α-helices and β-sheets, respectively. The nuclear localization signal (NLS) is located within the YABBY domain and has a complex nature, requiring the action of multiple amino acids within the YABBY domain and probably homodimerization of the proteins [40]. I–III, regions of variable amino acid sequences. Modified based on [40,41].

In 1999, Bowman and colleagues described a new family of genes encoding TFs that regulate a number of developmental processes and are unique for plants, the *YABBY* gene family [12]. Proteins encoded by the *YABBY* gene family members are characterised by the combination of two conserved domains: the classical Cys2/Cys2-type zinc finger domain and the basic helix-loop-helix (bHLH), called the YABBY domain [12,13] (Figure 2).

The zinc finger domain is located at the N-terminus of the YABBY protein. In the protein encoded by *CRABS CLAW (CRC*), the first identified *YABBY* gene from *Arabidopsis thaliana*, the zinc finger (from the 26th to the 53rd amino acid residue in the protein sequence) is formed by two α-helices surrounded by two β-folded layers [40] and represents a zinc chelating sequence. For *FILAMENTOUS FLOWER (FIL),* encoding another YABBY family TF of *A. thaliana*, it has been shown that four cysteine residues at positions 30, 33, 56, and 59 bind one zinc atom and are very important for protein function, while a proline at position 42 is located at the top of the “finger” structure [13]. For example, the *A. thaliana fil-2* mutation, where the guanine at position 89 is replaced by adenine, which results in replacement of cysteine for tyrosine at position 30 in the encoded amino acid sequence, leads to the loss of protein function [13].

The YABBY domain of the protein is structurally similar to the C-terminal part of the conserved HMG (high mobility group) protein domain [41] that is found in all eukaryotes and is composed of 80 amino acids that form three α-helices [42]. The sequence from amino acid position 146 to 179 of FIL protein is similar to the α-helix I and α-helix II of the HMG domain, but no homology between the YABBY domain of FIL and the canonical HMG domain has been found for the α-helix III [13]. Gross et al. [40] assumed that the YABBY domain (amino acid positions 109 to 155 in CRC) consists of one short and two long α-helices, while the sequence located between these domains (amino acid positions 54 to 108 in CRC) contains two β-folded layers, followed by a portion of unstructured amino acids that might serve as a mobile linker zone.

In angiosperms, the YABBY gene family consists of five gene subfamilies represented in *Arabidopsis* by six genes: *CRC*, *YAB1*/*YAB3* (*FIL*), *YAB2*, *YAB4* (*INO*), and *YAB5* [41,43]. A comparison of the structures of *YABBY* genes in *Arabidopsis*, *Cabomba caroliniana*, and rice (*Oryza sativa*) showed that all genes possess one intron in the same location in the region encoding the zinc finger-like domain, three introns in conserved locations in the region encoding the YABBY domain, and one intron in the non-conserved region between the two conserved regions [43,44]. A sixth intron is found in some genes in the non-conserved region downstream of the YABBY domain. The sixth intron is, however, consistently absent in all genes of the *YAB2* subfamily, which indicates that *YAB2* represents the ancestral form of *YABBY* gene family members [43]. In addition to the canonical conserved domains, proteins encoded by some members of the *A. thaliana YABBY* gene family (YAB1, YAB2, YAB3, and YAB5) have some similarity at the carboxyl end of the YABBY domain. In YABBY proteins, the amino acid sequence of the region located between the zinc finger and the YABBY domain is variable. Nevertheless, in YAB1, this sequence shows a certain similarity to that of YAB3, which confirms the viewpoint that the corresponding genes might have emerged as a result of the most recent duplications within the *YABBY* family [45]. In monocots, each protein of the YAB2 subfamily has a short conserved sequence of 15 amino acids located between the zinc finger and the YABBY domain [45].

## 3. Phylogeny of *YABBY* Genes

The *YABBY* gene family of *A. thaliana* comprises six members: *FILAMENTOUS FLOWER* (*FIL*, *YABBY1*, *YAB1*; AT2G45190), *YABBY2* (*YAB2*; AT1G08465), *YABBY3* (*YAB3*; AT4G00180), *YABBY5* (*YAB5*; AT2G26580), *CRABS CLAW* (*CRC*; AT1G69180)*,* and *INNER NO OUTER* (*INO*; AT1G23420). Based on phylogenetic analyses of YABBY proteins from multiple species, the corresponding proteins represent five monophyletic subfamilies, or clades of the YABBY family: FIL/YAB3, YAB2, YAB5, CRC and INO [43,46,47,48,49], which are named after the corresponding genes. Since representatives of these clades are found in all angiosperms, including the most basal (the ANA-grade members; [50]), it is assumed that at least five ancestral YABBY genes were present before the diversification of the extant angiosperms [21]. Whether these genes resulted from full-genome duplications or from duplications of shorter genomic fragments is still unclear. The closest paralogue of the CRC clade is the FIL/YAB3 clade, but the topology of the three remaining clades (YAB2, YAB5, and INO) is under debate [21,46]. According to one viewpoint, the INO clade clusters with FIL/YAB3 and CRC but branched off earlier in the course of evolution of the YABBY family [43]. Alternatively, the INO clade is nested within the same clade as YAB2 and YAB5 [21]. Since the relationships between the YAB2, YAB5, and INO clades and the cluster composed of the CRC and FIL/YAB3 clades are unclear, several possible scenarios have been proposed for the evolution of the YABBY family. One postulates that two simultaneous duplications of four ancestral *YABBY* genes resulted in a sister relationship between the YAB2 and YAB5 vs. the FIL/YAB3, INO, CRC clades [21,46]. The two other scenarios postulate that either the YAB2 or the YAB5 clade has a basal position [21,46]. In the course of angiosperm evolution, all YABBY subfamilies underwent various transformations. The strongest divergence occurred within the CRC, INO and YAB5 clades. The fact that most angiosperms have a single copy of *CRC* and of *INO* [46] led to the hypothesis that these subfamilies represent single lines of orthologues that diversified in the course of angiosperm speciation, not through ancestral duplication [48]. Conversely, the FIL/YAB3 and YAB2 subfamilies are represented in most flowering plants by numerous members with their numbers varying between monocots, basal angiosperms, and eudicots [46,51]. The *YABBY* genes of the basal angiosperm *Amborella trichopoda,* the *YAB2*-like genes in eudicots, and the *YAB2-*like genes of monocots cluster together as sister clades [46]. Seven *YABBY* members are present in the achlorophyllous parasitic plant *Monotropa hypopitys,* and each MhyYABBY belongs to one of four clades in the angiosperm YABBY family: *FIL*, *INO*, *CRC*, or *YAB5* [52]. A total of 12, 12, and 23 *YABBY* genes were identified in *Gossypium arboreum, G. raimondii,* and *G. hirsutum*, respectively, and the comparison of the sequences of the above cotton *YABBY* genes with those of the *YABBY* genes in Arabidopsis and rice showed that the N-terminal zinc-finger and the C-terminal YABBY domains in the YABBY proteins are highly conserved among cotton, Arabidopsis, and rice [51]. Monocots are characterised by an increased number of duplicated copies of genes from the *FIL/YAB3* and *YAB2* subfamilies and complete loss of *YAB5*; rice *YAB5* is part of the *FIL/YAB3* subfamily [44,46]. Twenty-one *YABBY* genes that were found in the *Triticum aestivum* genome can be sub-divided based on the structural similarities and functional differences of the encoded proteins into six subfamilies (YAB1/YAB3, YAB2, YAB5, CRC, and INO) [53].

Four clades of *YABBY* genes (A, B, C, D) have been revealed in three of the four orders of gymnosperms (Gnetales, Ginkgoales, and Coniferales), and the phylogenetic analysis of these genes indicates that at least four *YABBY* genes were present in the ancestor of extant gymnosperms [21]. Genes of the B clade are characterised by a 15 base pair deletion in the nucleotide sequence that encodes the YABBY domain. There are two possible scenarios for the origin of the *YABBY* genes in gymnosperms. According to the first scenario, the *YABBY* genes of all seed plants represent a monophyletic group originating from a single gene of the common seed plant ancestor and subsequently diverged independently in gymnosperms and angiosperms [21,46]. The alternative viewpoint considers the *YABBY* gene family as a paraphyletic group [21,43] with two *YABBY* genes present in the common ancestor of seed plants. Consequently, gymnosperm *YABBY* members are grouped with two clades of angiosperm *YABBY* sufamilies: the B and D clades are sister to the *CRC* and *FIL/YAB3* clades, while the A and C clades form a polytomy with the angiosperm subfamilies *YAB2, YAB5* and *INO* [21]. According to this topology, the two genes directing the development of angiosperm-specific “innovations”—the carpel (*CRC*) and the outer integument of the ovule (*INO*)—were derived from different *YABBY* gene ancestors.

## 4. Expression Patterns and Putative Functions of Members of the *YABBY* Gene Family

Based on the expression patterns in angiosperms, members of the *YABBY* gene family are divided into two groups, the so-called “vegetative” and “reproductive” genes [46,54]. The gene subfamilies *FIL*, *YAB3*, *YAB2* and *YAB5* are called “vegetative” *YABBY* genes because the members of the above gene subfamilies are expressed in cotyledons and leaves as well as in flower organs such as sepals, petals, stamens, and carpels, which evolved via modification of leaves, but are never expressed in the ovules [12,55,56,57,58,59]. In contrast, the group of “reproductive” *YABBY* includes *CRC* and *INO*, which are exclusively expressed in developing carpels and ovules, respectively [11,57,60]. Data about the expression of *YABBY* genes in diverse dicots and monocots are summarised in [61].

### 4.1. “Vegetative” YABBY Genes

**Figure 3:**
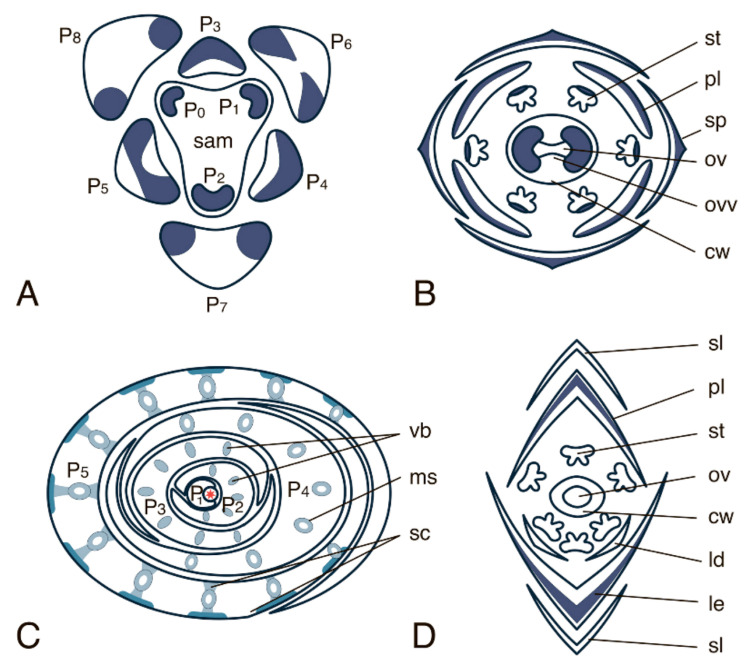
Expression pattern of the “vegetative *YABBY*” group. (**A**) Line drawing of the cross-section through the vegetative apex of *A. thaliana*. (**B**) Flower diagram of *A. thaliana*. (**C**) Line drawing of the cross-section through the vegetative apex of *O. sativa*. (**D**) Flower diagram of *O. sativa*. Patterns of “vegetative YABBY” expression are marked in blue colour and are based on descriptions and photos of in situ RNA-RNA hybridization from [44,55,57,63,64]. Here and in Figure 4, Figure 5 and Figure 6, sam—shoot apical meristem, P0-P7—successive stages of leaf primordia and leaf development, vb—vascular bundle, ms—mestome sheath, sc—sclerenchyma, sp—sepal, pt—petal, st—stamen, cw—carpel wall, ov—ovary, ovv—ovarian valves, lm—lemma, pl—palea, ld—lodicule, sl—sterile lemma (empty glume). The asterisk in (**C**) marks the SAM.

All “vegetative” *YABBY* genes in *A. thaliana* are similar in their overall expression patterns and differ mainly in the expression levels. The highest expression levels were detected for *FIL* and the lowest for *YAB2* [57]. *FIL* expression begins, in several subepidermal cells in the central part of the cotyledon primordia, during embryo development at the transition from the globular stage to the heart stage. At the early heart stage, *FIL* expression expands to the abaxial part of cotyledons, excluding their tips, and by the mid-heart stage, *FIL* expression marks the entire abaxial domain of the cotyledon. *FIL* expression persists in cotyledons during the torpedo, walking stick–shaped, and U-shaped embryo stages. Expression of *YAB2* and *YAB3* begins a little later, namely at the early heart stage, and their expression patterns are generally similar to that of *FIL*; the lower expression levels of *YAB2* and *YAB3* do not allow the detection of distinctive features. In *A. thaliana*, each of *FIL*, *YAB2,* and *YAB3* shows an expression pattern in leaves, which is similar to that in cotyledons: the expression is confined to a small group of subepidermal cells in the central part of the incipient leaf primordium, and in the cells of the future lower epidermis and the spongy mesophyll in the abaxial domain of the developing leaf. In the further course of leaf development, the expression of the above genes decreases in the central part of the abaxial domain, remaining rather high only at the leaf margin and switches off when leaf growth terminates (Figure 3A). This expression pattern led to the widely accepted assumption that the “vegetative” YABBYs are key regulators of the marginal meristem genesis. The expression of *YAB5*, similar to that of *FIL*, *YAB2,* and *YAB3*, marks the abaxial domain of the developing leaf. However, in contrast to *FIL*, *YAB2* and *YAB3*, *YAB5* shows the strongest expression in the main vein of the petioles and in the stem vasculature [55]. AtYAB3 has been shown to control vascular differentiation through suppression of xylem fate in the abaxial part of the leaf trace [62].

In *A. thaliana, FIL, YAB2,* and *YAB3* are also expressed in generally similar patterns in inflorescence, floral meristems, developing sepals, petals, stamens, and carpels [56,57]. As in leaves, the expression levels of *FIL* are the highest among those of the “vegetative” *YABBY* genes, and therefore the pattern of *FIL* expression is described in most detail. In the inflorescence meristem, *FIL* transcripts are first detected in the subepidermal cells of the flower primordium (FP); then *FIL* expression shifts to the abaxial domain of the flower primordium (FP). In the course of flower development, *FIL* expression first marks all subepidermal cells of the sepal and stamen primordia, but later translocates to the L1 and L2 of the abaxial side of the sepals and the central abaxial zone of the stamens (the future connective tissue) (Figure 3B). *FIL* is also expressed in several abaxial cell layers of the gynoecium primordium, from which the carpel wall is formed with highest expression levels detected in the cells of future ovarian mesophyll and the abaxial epidermis of the developing ovarian valves [57]. As the flower organs mature, the expression of *FIL*, *YAB2,* and *YAB3* decreases. None of *FIL*, *YAB2,* or *YAB3* is expressed in the roots of embryos or adult plants, or in ovule primordia [57].

*YABBY* genes have been identified in a wide range of angiosperms from diverse lineages and some of these genes were shown to be functionally similar to *A. thaliana YABBY* genes: expression of *YABBY* orthologues was observed in the leaves of *Bienertia sinuspersici*, which is a single-cell C4 plant [65]. Two orthologues of *AtYAB2*, which is essential for lamina outgrowth, were identified in cotton [51].

In the mycoheterotrophic plant *Monotropa hypopitys,* homologues of three *A. thaliana* “vegetative” *YABBY* genes (*MhyFIL1*, *MhyFIL2*, *MhyFIL3,* and *MhyYAB5*) were revealed. The transcripts of *MhyFIL1*, *MhyFIL2*, *MhyFIL3,* and *MhyYAB5* were identified in the bracts and flowers of *M. hypopitys*, which indicates that the encoded proteins, similar to their orthologues YAB1, YAB3, and YAB5, may influence the abaxial cell fate in all above ground lateral organs. However, in contrast to *A. thaliana*, in *M. hypopitys,* expression levels of *YAB5* were much higher than those of *FILs* [52]. Atypically for “vegetative” *YABBY* genes of other plants, *MhyYAB5* was expressed in roots. Therefore, *MhyYAB5* was hypothesised to control the development of the root system of *M. hypopitys*. Alternatively, the *MhyYAB5* expression in the roots may be explained by the fact that *M. hypopitys* roots bear underground adventitious buds, which presumably contain an embryonic inflorescence, which means that *MhyYAB5* could be expressed in the buds, not in the roots themselves [52]. At any rate, these data confirm the involvement of YABBY TFs in the regulation of the development of various leaf-derived organs. Bioinformatic methods have enabled the identification of homologues of “vegetative” *YABBY*s in the genomes of tomato (*Solanum lycopersicum* L., nine *YABBY* genes; [54]), apple (*Malus pumila* Mill., 13 *YABBY* genes; [66]), Chinese cabbage (*Brassica rapa* L. ssp. *pekinensis*; [67]), cucumber (*Cucumis sativus* L.; [68]), snapdragon (*Antirrhinum majus* L.; [69,70]), and grape (*Vitis pseudoreticulata,* two *YABBY* genes; [71]).

Members of all three gene subfamilies of the “vegetative” *YABBY* gene family (*CcFIL, CcYAB2* and *CcYAB5*) were found in the basal ANA-grade angiosperms *Cabomba caroliniana* and *Amborella trichopoda* [43,49,72]. *CcYAB5* is expressed in both vegetative and reproductive organs, i.e., in the abaxial domain of leaf primordia, the marginal cells at the tips of leaflet primordia and the procambium of leaf traces, the tips of the sepal- and petal-like leaflets of the simple perianth, and in the stamen primordia. However, the highest expression levels mark the cells of carpel primordia that subsequently differentiate into the placenta [43]. As the carpels develop, the expression levels decrease, but the transcripts can still be detected at the time of ovule initiation [43,49]. *CcFIL* expression marks the procambial strands in flower primordia, the epidermis, and mesophyll of the sepal- and petal-like perianth leaflets, whereas no expression of *CcFIL* is detected in placenta tissues. Expression levels of *CcYAB2* are very low; transcripts thereof were detected in flower primordia only by semi-quantitative RT-PCR [43]. Mutually exclusive expression patterns of *CcYAB5, CcFIL,* and *CcYAB2* in *C. caroliniana* and their homologues in *A. trichopoda* led to the hypothesis that although *YABBY*-mediated regulation in basal angiosperms is generally similar to that in most other flowering plants, the *FIL/YAB5* genetic pathway has undergone certain modifications in the course of angiosperm diversification [43]. Supposedly *YAB2* and *YAB5* are among the regulatory genes associated with the radial morphology of the stamen filament in many angiosperms [45,73].

The model species for the study of YABBY family transcription factors in monocots is rice (*Oryza sativa*), the genome of which contains seven “vegetative” *YABBY* genes: *OsYABBY1* (*OsYAB1*)*, OsYABBY2* (*OsYAB2*)*, OsYABBY3* (*OsYAB3/TOB3*)*, OsYABBY4* (*OsYAB4/TOB2*)*, OsYABBY5* (*OsYAB5/TOB1*), *OsYABBY6* (*OsYAB6*), and *OsYABBY7* (*OsYAB7*) [44]. In contrast to the overall conservation of expression patterns of “vegetative” *YABBY* genes in several dicots examined, the expression of those genes in grasses is characterised by certain peculiarities. For instance, expression of *OsYABBY1* is not detected in incipient leaf primordia, but begins approximately at the P2 stage and is restricted to the future large vascular bundles, with the highest expression levels at the stage of the differentiation of phloem and metaxylem (P3-P4) [44]. By stage P4, *OsYABBY1* expression becomes restricted to the future mestome sheath of the vascular bundle, and at the P5 stage, it expands to the sclerenchyma with the highest expression levels in sclerenchyma cells located abaxially to the leaf bundle (Figure 3C). In rice spikelets, *OsYABBY1* is expressed in the central and basal parts of both lemma and palea primordia. At early stages of flower development, *OsYABBY1* expression does not have abaxial polarity, but later the gene expression becomes confined to the future sclerenchyma cells located in the central and abaxial parts of lemma and palea (except for the entire epidermis) (Figure 3D). Very weak and scattered expression of *OsYABBY1* sometimes marks primordia of stamens and carpels of developing spikelets in early developmental stages [44,63]. Studies suggest that in rice, TFs encoded by the orthologues of “vegetative” *YABBY* genes *FIL/YAB3*, *YAB2*, and *YAB5* act as homo- and heterodimers as the loss of the *YAB5* genes could be complemented by the *FIL* and *YAB2* paralogues [44,46].

Recently 17 genes from the *YAB2* subfamily and 14 genes from the *FIL* subfamily were identified in the genome of an orchid, *Phalaenopsis equestris*. Expression of three genes from the *YAB2* subfamily (*PeYAB2*, *PeYAB3*, and *PeYAB4*) was detected in both vegetative and reproductive tissues [74]. *YABBY* homologues were also found in *Ruscus aculeatus* [75] and *Lilium longiflorum* [76]. It was suggested that YABBY homologues in monocots do not regulate organ polarity, but instead regulate leaf morphogenesis and the maintenance of the floral meristem; this can be considered an argument in support of the hypothesis that OsYABBY1 may have been recruited to regulate the development of grass-specific tissues and organs during evolution [44].

Together with the results of the functional analyses, these data led to the hypothesis that vegetative YABBYs regulate a broad lamina-specific genetic programme involving the repression of SAM identity and maintenance genes (*WUSCHEL* and *class I KNOX*) while promoting expression of genes that are the polarity and lamina maturation markers [55,77,78]. The YABBY TFs were hypothesised to act in concert with *AINTEGUMENTA* (*ANT*, encoding an AP2/ERF family transcription factor), as the *fil ant* and the *yab3 ant* double mutants have reduced lamina growth compared to the corresponding single mutants [79]. During the initiation of primordia of simple leaves and flowers, *A. thaliana* YABBY TFs interact with auxin response factors (ARFs) and a YABBY/ARF complex silences *STM*, which is a *class I KNOX* gene, via histone deacetylation [80]. It has also been suggested that acting from leaf primordia, YABBY TFs promote SAM maintenance via an unknown signal [20,64], consistent with the observation that some “vegetative” YABBY TFs regulate shoot apical meristem development and phyllotaxy [14,44,55,77].

Determination of leaf polarity is complex. Adaxial-abaxial polarity is established in the PZ prior to leaf primordium formation [16]. The gene encoding the KANADI transcription factor KAN1, an abaxial determinant, is expressed in a ring around the SAM. In combination with the auxin response factor ARF4, KAN1 upregulates the expression of the *FIL* and *YAB3* genes, while the FIL and YAB3 TFs stimulate the expression of *ARF4* and *KAN1* [14,81,82]. Together with the Lateral Organ Boundaries (LOB) TF AS2, the adaxial determinant ARP represses the expression of *YAB5*, *KAN2*, *ARF3,* and *ARF4*, while stimulating the expression of another group of adaxial determinants, the HD-ZIPIII TFs. The polarisation of leaf primordia is required for their further development [83,84,85]. ARP/AS2 repress the transcription of KAN1 and ARF3/4, while KAN1-ARF3/4 repress the transcription of ARP/AS1 and the HD-ZIPIIIs. The YABBY TFs are not required for the initial establishment of leaf polarity, but rather are involved in initiating lamina outgrowth and polarity maintenance.

At the boundary between the adaxial and abaxial leaf domains, FIL/YAB3 TFs upregulate the expression of the *WOX1* and *WOX3* genes that redundantly specify lateral lamina outgrowth [86]. Thus, *YABBY* together with the *WOX* genes determine growth along the medio-lateral axis [87]. Both YABBY and WOX TFs regulate lamina outgrowth in a non-cell autonomous manner in that they can affect the differentiation of tissues that lack their expression [55,57,77,86,88], possibly via the generation of a mobile signal, e. g. a phytohormone. It was proposed that *YABBY* genes control auxin flux and auxin response along the leaf margin as a *Arabidopsis* quadruple *yabby* mutant shows perturbed venation and lack of marginal growth [55]. “Vegetative” YABBY genes were also shown to act as transcriptional activators of jasmonate-triggered responses [89]. Consistent with a role in the jasmonate response, recent studies have revealed some new functions of YABBY TFs in the response to abiotic stress (salt, heat, and drought stress response) in pineapple, soybean, grapevine, pomegranate, and cotton [51,61,90,91,92,93], leaf curling [94,95], and in the delay of the flowering stage [95].

In summary, the “vegetative” *YABBY* genes play an important role in the initiation and development of leaves and leaf-derived organs in diverse angiosperms. They suppress the expression of meristem-specific genes in leaf primordia, regulate the establishment of dorsoventral leaf polarity, initiation of the marginal meristem, and phloem differentiation in leaf veins [14,55,57,58,69,70,77,96]. Furthermore, in dicots, the “vegetative” *YABBY* genes play a key role in formation of the marginal meristem, from which leaf blade and stipule are originated. However, in monocots, “vegetative” *YABBY* genes are likely not involved in the determination of polarity nor in regulating the marginal meristem, but rather control the development of vascular bundles, mestome sheath, and sclerenchyma. The expression of “vegetative” *YABBY* genes in both leaves and flower organs is thought to reflect the foliar evolutionary origin of the latter.

### 4.2. The “Reproductive” YABBY Genes

#### 4.2.1. *CRC* Gene Subfamily

**Figure 4:**
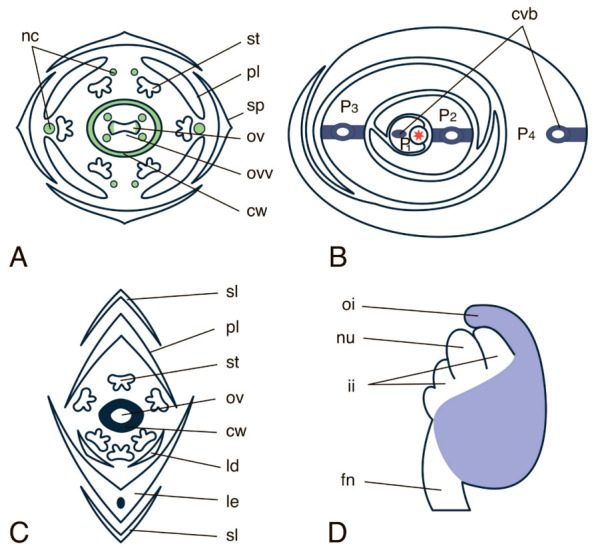
Expression pattern of the “reproductive” *YABBY*” group. (**A**–**C**) Expression of *CRC* subfamily, (**D**) expression of *INO* subfamily. (**A**) Flower diagram of *A. thaliana*. (**B**) Line drawing of cross section through vegetative apex of *O. sativa*. (**C**) Flower diagram of *O. sativa*. (**D**) Ovule of *A. thaliana*. Schemes are based on descriptions and photos of in situ RNA-RNA hybridizations from [12,19,60,64,97,98]. cvb—central vascular bundle, nc—nectary, oi—outer integument, ii—inner integument, nu—nucellus, fn—funiculus, asterisk in (**B**) marks the SAM.

The *CRC* gene of *A. thaliana* is expressed in developing flowers with the highest expression level in carpels and nectaries. The transcription of *CRC* in carpel primordia is confined to the two domains: the outer epidermal cells around the circumference and the inner cells of the future valve regions and the four internal patches. No *CRC* expression is observed in cells that give rise to the placenta and septum (Figure 4A) [12]. During fruit development, *CRC* expression in the placental tissues, in the future silique septum, its valves and in the ovules gradually decreases in the basipetal direction starting from the apical domain. Low levels of *CRC* expression mark the central cells of the sepal and petal primordia and the group of receptacle cells adjoining sepals at the early stages of their development, but *CRC* expression becomes turned off by the time of inception of stamens and carpels [12]. Thus, *CRC* expression is not strictly confined to the abaxial tissues of the flower organ primordia. *CRC* is supposed to play a key role in the development of nectaries because expression of *CRC* starts at the moment of inception of flower organ primordia and persists until the pollination stage, and because *CRC* mutants do not develop any nectaries [12]. Another putative *CRC* function is the control of flower meristem determinacy: knockout mutants form more carpels than the wild-type plants, supposedly because the meristem in the knockout mutants becomes indeterminate [12]. *CRC* is also required for carpel fusion [40].

Numerous *AtCRC* homologues found in early divergent angiosperms, i.e., angiosperms which diverged before the dicot-monocot divergence, seem to have similar functions. For example, the expression of the *CRC* homologue from the mycoheterotrophic plant *M. hypopitys*, *MhyCRC,* is confined to flower tissues, which suggests *MhyCRC* plays a conserved role in carpel fusion, style/stigma, and nectary development, and in the determinacy of the floral meristem [12,52,99,100]. However, in “basal eudicots”—i.e., in several paraphyletic lineages at the base of the eudicots that tend to show ancestral characteristics—*CRC* homologues in basal eudicots have some additional features as compared to those in “core eudicots”—i.e., in the monophyletic eudicot group traditionally termed tricolpates or non-magnoliid dicots. Specifically, although the expression pattern of the *CRC* orthologue in the female flowers of the basal ANA-grade angiosperm *Amborella trichopoda* is similar to that in other angiosperms, *AmbCRC* transcripts are also found in the staminate filaments of the male flowers [72]. In the basal eudicot *Eschscholzia californica* (Ranunculales), the *CRC* orthologue is expressed throughout flower development, but at anthesis, expression of *CRC* is restricted to the gynoecium [101]. At early stages of gynoecium development, *EcCRC* mRNA is localised in the abaxial domain of the carpel wall and in the center of the carpel base, while at later stages, expression of *EcCRC* expands to the medial part the carpel wall with the highest expression levels in the cells adjacent to the future placenta [101]. Although the *EcCRC* transcription is downregulated in developing ovules, its virus-induced suppression leads to a number of aberrations in ovule development, which suggests that CRC plays a role not only in the development of carpel and placenta, but also in ovule initiation [101].

*CRC* orthologues have been found in a number of monocots (*Oryza sativa, Triticum aestivum, Sorghum bicolor*, and *Zea mays*), and based on the phenotypes of the corresponding mutants, the above *CRC* ortologues have collectively been named *DROOPING LEAF* (*DL)* [97,102,103]. The amino acid similarity of *O. saliva* DL with the other YABBY proteins spans from 48.6 to 87.5% within the zinc finger domain and from 62.7 to 83.6% within the YABBY domain [103], while the amino acid similarity between different DL proteins lies within 78–95% [102]. Unlike *CRC* in dicots, *DL* in *O. sativa* is expressed in both reproductive and vegetative organs, and the silencing of this gene affects the development of both flowers and leaves [97,103]. *OsDL* expression starts at the P1 stage of leaf development, where it marks the central zone of the leaf primordium [97,103]. At later stages of leaf development (stages P2-P3), *OsDL* expression becomes restricted to several bands of cells between the adaxial and abaxial epidermis. By stage P4, *OsDL* transcription is confined exclusively to mesophyll cells located abaxially from the central vascular bundle (Figure 4B). In double *dl* and *sup1* (SUPERMAN1, a TF that prevents stamen formation in whorl 4 of a flower) knockout mutants, the number of cells in the central part of the leaf is significantly reduced as compared to that in wild-type plants. Therefore, it was suggested that DL TFs induce cell proliferation along the dorsoventral axis of the central leaf domain [103]. Since *DL* is expressed exclusively in the central domain of the leaf, and its overexpression does not cause any changes to the dorsoventral leaf structure, DL is more likely to regulate medial-lateral than abaxial-adaxial patterning [103]. Single recessive mutations of *DL* homologues in a number of monocot species (*Pennisetum glaucum, Hordeum vulgare,* and *Panicum maximum*) cause similar aberrations in development of the gynoecium and the main leaf vein, leading to drooping leaves, the basis of the so-called *DROOPING LEAF* phenotype, which is unique for monocots [104]. In monocots, *DL* transcription in the floral meristem is confined to the zone where carpels are initiated, marks the carpel wall in the course of its development, but is excluded from the central meristem domain where the single ovule is formed after the carpel fusion. *DL* is also expressed in the central vein of the lemma (Figure 4C) [97,103]. Thus, DL TFs, similar to other CRC homologues, likely regulate the development of the carpel wall, but not that of the ovule. Similarly to CRC in dicots, DL also regulates determinacy of the floral meristem, i.e., when *DL* is silenced, a greater number of carpels is formed, compared to that of wild-type plants [97]. In the orchid *Phalaenopsis equestris,* 15 homologues of *CRC/DL* were identified, two of which were found to be expressed in developing floral buds (stage 1) and in the gynostemium (the fused male and female reproductive organs) as well as at early developmental stages of the ovule [74].

In summary, in different angiosperm species, the TFs encoded by the *CRC* gene subfamily regulate a wide range of different developmental processes: determinacy of the floral meristem, longitudinal, and radial growth of the gynoecium, ovule initiation, establishment of carpel wall polarity and development of the future fruit septum. Since *CRC* expression is upregulated in meiocytes in dicots and monocots [105], it is assumed that the protein encoded by the ancestral angiosperm *CRC* gene—acquired by angiosperms before the divergence of dicots and monocots—had two main functions. The first (inferred based on the comparison of the expression patterns of *CRC* genes in an evolutionary context and on the analysis of *crc* knockouts) is regulation of carpel development and specification of the abaxial gynoecium domain in the predecessor of modern angiosperms. The second plausible function of the protein encoded by the ancestral *CRC* gene is the regulation of the switch, during flower development, from indeterminate growth of the floral meristem to determinate growth thereof. In some groups of flowering plants, *CRC* homologues have gained specific new functions, i.e., functions in genesis and development of both floral and extrafloral nectaries specifically in basal dicots [48], differentiation of the main vein tissues, and specification of carpels in monocots [103]. Recent studies suggest that *CRC* homologues are involved in regulation of vascular development in *Pisum sativum* and *M. hypopitys* [52,106].

#### 4.2.2. *INO (INNER NO OUTER)* Gene Subfamily

Although the expression of *Arabidopsis thaliana INO* starts at the globular stage of embryogenesis, its role in embryo patterning is not known yet. However, the role of *INO* during the generative phase of ontogenesis is well established: *INO* regulates development of the outer ovule integument [52,60,107,108,109,110]. *INO* expression during ovule development has been described in detail for a wide range of both basal and early divergent angiosperms. Surprisingly, the expression patterns of *INO* orthologues are similar across plants that differ in the structure of integuments [111].

In the anatropic bitegmal ovules of *A. thaliana*, *INO* expression starts in several cells on the abaxial side of the chalaza and expands first to the epidermal and subepidermal cells at the abaxial side of the ovule primordium and later to the site of the initiation of the outer integument [60,112,113]. Following the development of the outer and inner integuments, the *INO* expression marks the abaxial side of the outer integument (Figure 4D). In the course of ovule maturation, the *INO* transcription becomes restricted to the chalazal zone of the outer integument.

Some of the *YABBY* homologues identified in cotton are also expressed in ovules, which suggests that these genes play a role in the regulation of ovule development [51]. Two *INO* homologues identified in the mycoheterotrophic plant *M. hypopitys* (*MhyINO1* and *MhyINO2*), similarly to *A. thaliana INO*, seem to promote growth of the outer ovule integument [52,60,108]. The expression pattern of an *INO* homologue in the orchid *Phalaenopsis equestris* also suggests a role in the development of the outer ovule integument [74].

Altogether, since silencing of the *INO* (*INNER NO OUTER*) subfamily genes always led to the lack of an outer integument of the ovule, and these genes are expressed specifically on the abaxial side of the outer integument of the ovule, the *INO* genes are thought to be among key regulators of ovule development [60,107]. The asymmetry of the *INO* expression—its confinement to the abaxial side of the developing ovule axis—is thought to determine the correct location of the outer integument, while the *INO* expression in the abaxial layer of the outer integument establishes its polarity, which is a prerequisite for development of any lateral organ [113]. Since the polarity in the *INO* expression and, as a result, the polarity of the outer integument is found in early divergent angiosperms, the polarity of the outer integument is considered ancestral for all living angiosperms [110].

Consistent with this hypothesis, the *INO* orthologues from the early divergent angiosperms *Nymphaea alba* and *Annona squamosa*, are expressed in the cells of the abaxial epidermis of the outer integument [107,110]. However, *NaINO* is also expressed in the cells of inner integument and the tip of the nucellus of *N. alba* [110]. Based on these data and on the expression patterns of *INO* homologues, in the early divergent angiosperms *Cabomba caroliniana* and *Amborella trichopoda*, in other flower parts, Yamada et al. [43] concluded that the carpel-specific expression of *INO* dated back to a time point after divergence of the Nymphaeales.

### 4.3. Comparison of YABBY Genes in Different Groups of Plants

#### 4.3.1. *YABBY* Genes in Angiosperms

Although the expression patterns of various “vegetative” and “reproductive” angiosperm *YABBY* genes vary, these genes are usually expressed in the abaxial domains of organs. Analyses of early divergent angiosperms suggest that in the common ancestor of extant angiosperms, the *YABBY* genes were expressed at least in the abaxial domains of lateral organs [43], while the abaxial expression of at least one *YABBY* gene in both leaves and reproductive organs of leaf origin seems to be a common feature of dicotyledonous plants [57,113]. Abaxial expression of both “vegetative” *YABBY* genes (*FIL*, *YAB2,* and *YAB5*) in developing leaves and their “reproductive” counterparts (*CRC* and *INO*) in various floral organs is thought to indicate the common evolutionary origin of leaves, lateral floral organs, and the outer integument in angiosperms [1,113]. Expression of “vegetative” *YABBY* homologues in primordia of both vegetative and reproductive organs in the basal angiosperms *Cabomba caroliniana* and *Amborella trichopoda* suggests that the ancestral *YABBY* gene regulated cell proliferation in all lateral organs [43,49,72]. Therefore, the ancestral *YABBY* genes are suggested to have been involved in the regulation of leaf blade development and to have played a crucial role in the establishment of leaf dorsoventral symmetry in the course of megaphyll evolution in seed plants [35,55]. It has been revealed that during transition to flowering, both “vegetative” and “reproductive” YABBY TFs are required to establish a correctly developing flower primordium through interaction with other TFs (REV, KAN4, SEU, LUG, ANT, SUP, LFY and the TFs encoded by the floral homeotic MADS-box genes, AP3, PI, SEP; [13,58,79,109,114,115]). *CRC* expression was shown to be upregulated by AP3/PI/SEP TFs, to be involved in the control of radial and longitudinal gynoecium growth, carpel fusion and nectary location, and to participate in the determinacy of the floral meristem through repression of *WUS* expression [12,47,99,101]. The question of whether *INO* and *CRC* originated from one or from different ancestral *YABBY* genes, which are responsible for development of structures unique to flowering plants, carpels (*CRC*) and integuments (*INO*), has not been resolved yet [46]. Since the outer integument is a synapomorphy of angiosperms, it should have emerged simultaneously with divergence of *INO* genes from the ancestral *YABBY* genes [107,110].

#### 4.3.2. *YABBY* Genes in Gymnosperms

Four clades of *YABBY* homologues (A, B, C, and D) were found in gymnosperms from the orders Gnetales, Ginkgoales, and Coniferales. The phylogenetic analyses of the above genes suggest that either one or two *YABBY* genes were present in the last common ancestor of extant seed plants [21].

**Figure 5:**
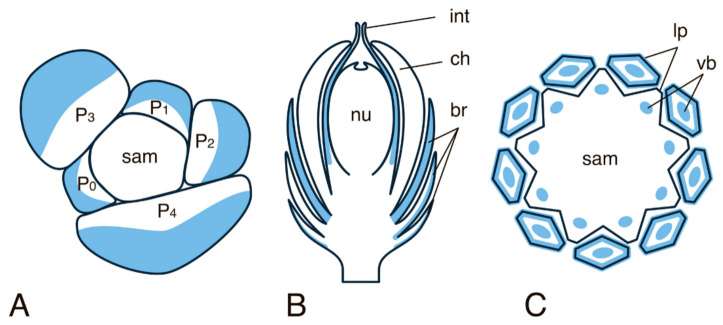
Expression pattern of *YABBY* homologs in gymnosperms. (**A**) Line drawing of the cross-section through the vegetative apex of *Gingko biloba.* (**B**) Longitudinal section through the ovule of *E. distachya.* (**C**) Line drawing of the cross-section through the vegetative apex of *Pseudotsuga menziesii.* Schemes are based on descriptions and photos of in situ RNA-RNA hybridizations from [21,116]. lp—leaf primordium, int—integument, br—bract, ch—chlamys.

Similarly to the “vegetative” *YABBY* genes in angiosperms, three *YABBY* homologues from *Ginkgo biloba* (Ginkgoales), *GbiYABC*, *GbiYAB1B,* and *GbiYAB2B* are expressed in the abaxial domain of leaf primordia and the abaxial domain of young leaves (Figure 5A) [21]. However, unlike their angiosperm counterparts, the above *YABBY* homologues are also expressed in differentiating leaf vascular tissues. Expression of two of the three *YABBY* homologues in developing leaves takes place in a complementary manner: *GbiYAB1B* is transcribed on the abaxial side of the leaf base, while *GbiYAB2B* expression is confined to the abaxial domain of the leaf tip. Thus, they seem to represent paralogues formed as a result of duplication followed by subfunctionalisation [21].

Two *YABBY* homologues found in *Ephedra distachya* (Gnetales), *EdiYABB,* and *EdiYABD,* are expressed in the female megastrobili. *EdiYABD* mRNA is localised both in the single integument of the ovule and in the scale-like sterile leaves (bracts) (Figure 5B). In the inner whorl, *EdiYABD* is expressed in the entire bracts, while in the outer whorl, its expression is confined to their abaxial side. *EdiYABB* is expressed in the outer layer of the ovule integument and the base of the bracts; the expression levels are higher on the abaxial side. It is likely that while in angiosperms, the developmental programs regulated by the “vegetative” and “reproductive” *YABBY* genes, respectively, differ, at least in some gymnosperms, the same TFs control development of both the leaf and ovule integument [21].

Since *YABBY* homologues are expressed, in both leaves and female cones of Gnetales and Ginkgoales, in a polar abaxial manner with a pattern similar to the expression patterns of *YAB2*, *YAB5*, and *FIL/YAB3* in angiosperms, Finet et al. [21] speculated that *YABBY* genes already acted on leaf polarity in the last common ancestor of extant seed plants. However, in young needle-like leaves of the conifer *Pseudotsuga menziesii, PmeYABC* is expressed in the provascular tissues of leaf primordia and differentiating vascular bundles without any abaxial/adaxial polarity (Figure 5C). The foregoing suggests that while polar *YABBY* expression can promote the development of leaves with a wide lamina, e. g. leaves found in Gnetales and Ginkgoales, this is not the case in Coniferales [21].

Thus, the expression patterns of the *YABBY* homologues in Gymnosperms and the expression patterns of their angiosperm counterparts have both similarities and differences.

#### 4.3.3. YABBY Genes in Seedless Plants

Since *YABBY* homologues had not been found in the genomes of the liverwort *Marchantia polymorpha* [24], the bryophyte *Physcomitrella patens* [117], the lycophyte *Selaginella moellendorffii* [23], and the pteridophytes *Azolla filiculoides* and *Salvinia cucullata* [118], YABBY TFs were considered unique for seed plants [21,26,119]. However, a *YABBY* gene precursor was found in the green alga *Micromonas* [39], and *YABBY* homologues were identified in transcriptomes of the homosporous lycophytes *Huperzia selago and Huperzia serrata* (Lycopodiales) [36,37] and lately in the genomes of three hornwort strains of the genus *Anthoceros* [38]. These findings indicate that the last common ancestor of hornworts and seed plants already contained a YABBY-domain protein and also suggests that YABBY TFs, like other regulators of *KNOX* gene expression, evolved in algae.

**Figure 6:**
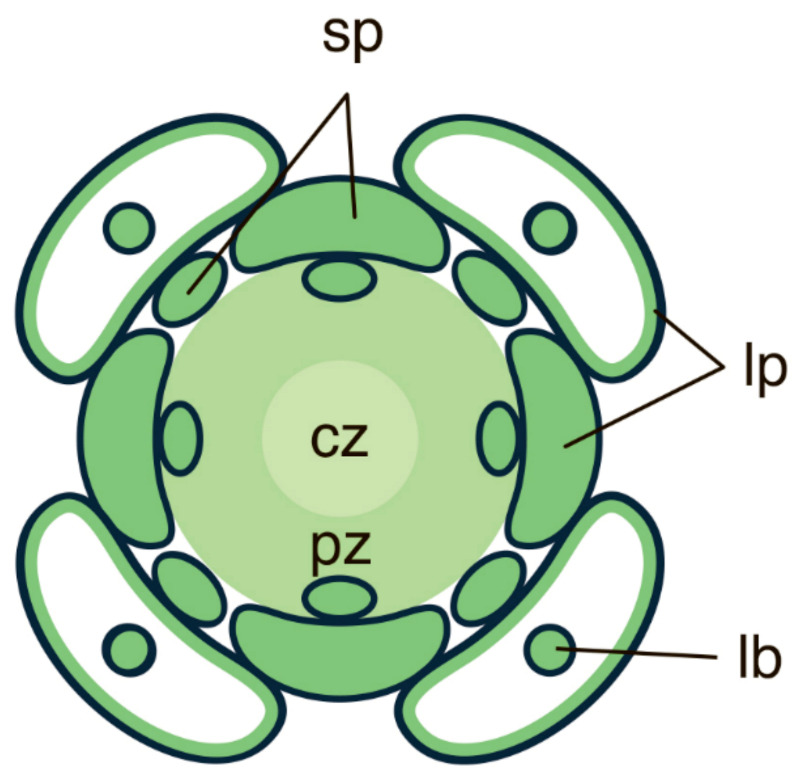
Expression pattern of *YABBY* homologs in lycophytes. Scheme is based on descriptions and photos of in situ RNA-RNA hybridizations from [36]. cz—central zone of the SAM, pz—peripheral zone of the SAM, lp—leaf primordium, sp—sporangium.

One member of the *YABBY* gene family has so far been found in *Huperzia selago.* This gene is referred to below as *HsYABBY*. Expression of *HsYABBY* in the lycophyte *H. selago* and expression of *HsYABBY* counterparts in seed plants have showed several major differences. First, *HsYABBY* expression marks all cells of the leaf primordia without any polar distribution [36]. This expression pattern resembles the expression of *YABBY* genes in the leaves of the conifer *Pseudotsuga menziesii* [21] and suggests that, similarly to what has been observed in needle-like conifer leaves, YABBY TFs do not act as regulators of leaf polarity in the microphylls of lycophytes. Second, *HsYABBY* expression marks not only primordia of leaves but also primordia of sporangia, another type of lateral organs originating from the SAM. This supports the scenario that at least in lycophytes, leaves could have originated in the course of modification of the sporangia developmental programme [8]. Third, *HsYABBY* expression is confined not only to the primordia of lateral organs but also to the peripheral cells of the SAM [36], while the expression of the *YABBY* genes in angiosperms is always excluded from the SAM. This difference in the expression suggests that in early divergent land plants, YABBY TFs might have been involved in both regulation of organogenesis and control of SAM maintenance (Figure 6).

Altogether, these findings suggest that *YABBY* genes have already played a role in regulation of organogenesis in lycophytes, where their functions neither involved repression of SAM indeterminacy nor establishment of the leaf polarity. At first glance, the expression of *YABBY* genes in the SAM contradicts a well-known function of YABBY TFs, i.e., suppression of cell indeterminacy [41]. Nevertheless, in angiosperms, *YABBY* genes are recognised as key regulators in establishment and subsequent functioning of marginal meristems, which are specific meristematic tissues on the leaf margin [35,55,120]. Moreover, in the compound leaves of the primitive angiosperm *Cabomba caroliniana, YABBY* genes were shown to regulate the formation of leaflets, i.e., the local initiation of cell proliferation [49,72]. Thus, *YABBY* TFs in angiosperms can stimulate cell proliferation, at least as far as the regulation of planar, not radial, growth is concerned. Since KNOX TFs have also been identified in *H. selago* [36], it is possible that at least in Lycopodiaceae, YABBY and KNOX TFs could cooperatively maintain cell proliferation both in the SAM and in the primordia of lateral organs, namely leaves and sporangia. In the course of subsequent duplication and subfunctionalisation of these TFs during land plant evolution, KNOX TFs could have taken control over the maintenance of apical meristems, and YABBY TFs over the establishment of marginal meristems.

The expression pattern of *HsYABBY* in both SAM and leaf primordia of *H. selago* (Lycopodiales) [36] resembles the expression pattern of another gene, *ARP,* which regulates leaf development in the lycophyte *Selaginella kraussiana* (Selaginellales) [28]. This similarity of the expression pattern of *HsYABBY* and that of *ARP* as well as the absence of YABBY TFs in *S. kraussiana* and the absence of the ARP TFs in *H. selago* seems to suggest that the common ancestor of homosporous and heterosporous lycophytes probably contained both transcription factors. Later, the *ARP* gene or its expression in shoot apices was lost in Lycopodiales, while in Selaginellales, the *YABBY* gene was lost [36]. This difference in molecular regulation of leaf development between the two lycophytes (*YABBY* vs. *ARP*) might be correlated with the differences in the structural type of their SAMs. Like most other seedless vascular plants (ferns), plants of Selaginellales have an SAM with a single apical initial, i.e., a monoplex SAM. This SAM type is characterised by an almost invariant pattern of cell divisions of the single apical cell and its derivatives. In a monoplex SAM, both microphylls and megaphylls are initiated through the establishment of new apical cells (initials) in parts of the surface layer of the SAM [121,122]. In *H. selago*, like in other Lycopodiales, the SAM is of simplex type, i.e., the SAM has several apical initials, which is unusual for seedless plants. In *H. selago*, the SAM is characterised by a cytohistological zonation, wherein a group of bigger and more vacuolated cells is in the center (the central zone, or CZ) and a group of smaller less vacuolated cells is at the periphery (the peripheral zone or PZ). While in the monoplex SAM organogenesis is achieved through establishment of new apical initials, in the simplex SAM primordia of both leaf and sporangia originate via proliferation of a group of surface and subsurface cells of the PZ. Thus, the simplex SAM organogenesis in *H. selago* resembles such organogenesis in seed plants. Traditionally, it has been assumed that the ancestral SAM type was monoplex, and the simplex SAM of lycophytes and that of gymnosperms are homoplasious and originated independently from each other (discussed in [123]). However, recent transcriptome analyses have revealed that the transcript patterns of the monoplex SAMs of lycophytes and ferns show more similarity with those in the SAM of the angiosperm *Zea mays* than with each other, suggesting that monoplex SAMs evolved independently in both lineages, and that the ancestral SAM type was simplex [124]. Additional support for this hypothesis comes from the fact that the lycophyte *S. kraussiana* with a monoplex SAM contains an *ARP* gene [28], while the fern *Ceratopteris richardii* with the same SAM type does not contain this gene [34].

Consequently, simplex, but not monoplex, SAMs might be plesiomorphic for land plants [36,124]. This hypothesis was first proposed by Kidston and Lang [125] based on the fact that the SAM of the extinct genus *Rhynia*, one of the most primitive land plants, is characterised by multiple apical initials. According to one scenario, the key factor that caused the independent reversal to the monoplex SAM in heterosporous lycophytes and most ferns was the loss of the ability to establish intercellular communication between cells post-cytokinetically by the development of so-called secondary plasmodesmata (PD) [36,123,126]. As a result, in these plants, intercellular communication remained possible only through plasmodesmata formed during cytokinesis, the so-called primary PD, and only the monoplex SAM structure, where all cells are derived from the single apical cell (AC), can enable intercellular contacts between all cells via primary PD [36,123,126]. Given that YABBY TFs must have been present in the common ancestor of land plants [36] and have a function in the suppression of the expression of *class I KNOX* genes that encode non-cell-autonomous TFs [12,13,55], we hypothesize that they were preserved in the ancestral simplex SAM of the first land plants, while the modifications associated with the independent reversal of the simplex SAM to the single AC SAM type in some lycophytes and most ferns included the loss of YABBY-controlled regulation of leaf development.

## 5. Conclusions

With the rapid accumulation of genomic and transcriptomic data on different representatives of the plant kingdom, it becomes evident that the genetic networks that are known to regulate morphogenesis of land plant sporophytes with indeterminate apical growth and three-dimensional bodies were most likely co-opted and modified from the genetic programs that were already present in green algae (Chlorophyta and/or Charophyta) and gametophytes of bryophytes *sensu lato* that predated plants of the sporophytic evolutionary line [119,127]. Among the transcription factors that regulated these co-opted genetic programs in green algae are KNOX/BELL heterodimers that control the switch between the haplo- and diplophase, i.e., the progression through the life cycle [127,128], class I KNOX TFs that are involved in the establishment of transient apical growth in moss sporophytes, and class II KNOX TFs that repress gametophytic programmes in bryophyte sporophytes [129,130] as well as *CLAVATA* homologues that are responsible for the transition from planar (two-dimensional) to three-dimensional growth in the gametophytes of mosses [131].

YABBY TFs used to be considered unique for seed plants [21,26,119]. However, the presence of a *YABBY* gene precursor in the green alga *Micromonas* [39], and of *YABBY* homologues in the sporophytes of hornwort *Anthoceros* spp. [38] and in transcriptomes of the homosporous lycophytes *Huperzia selago and Huperzia serrata* (Lycopodiales) [36,37] led to the conclusion that YABBY-domain proteins were present in the last common ancestor of land plants. In combination with the fact that *KNOX* homologs, encoding TFs important for shoot morphogenesis, are present in streptophyte algae [119,127,128], bryophyte sporophytes [129,130], and lycophytes [33,36], these data suggest that YABBY TFs, like other regulators of *KNOX* gene expression, evolved in algae. A hypothesis says that the ancestral function of *class I KNOX* genes was the gradual postponement of meiosis that has led to the prolongation of the diplophase and resulted in the emergence of a multicellular diploid sporophyte [119,128]. In combination with the fact that the *YABBY* homolog is expressed both in the SAM and in the sporangia primordia in the lycophyte *H. selago,* this enables the speculation that KNOX TFs together with YABBY TFs could have combined the regulation of the switch between haploid and diploid programmes with the regulation of cell proliferation in the diplophase.

The emergence of leaves, lateral appendages with limited growth and flattened blades that have enormously increased the efficiency of photosynthesis and turned out to be essential for life on land, was a milestone in the evolution of sporophytes of early land plants. Leaves could have resulted from the modification of genetic programmes that likely already existed in the gametophytes of bryophytes and/or in sporophytes of the first leafless land plants (rhyniophytes *sensu lato*). Among the TFs that could have been recruited to regulate this new developmental programme are the ARP homologues are expressed in both leaves and shoot apices of some lycophytes and ferns; therefore, ARP TFs were considered candidates for controlling shoot dichotomy in early leafless land plants [28]. This scenario is consistent with the so-called telome theory [3] that suggests that leaves originated from gradual modification of dichotomising leafless axes (so-called telomes) of rhyniophytes *sensu lato*. In this framework, the next step in leaf evolution was the so-called “planation”, which is the reversion to two-dimensional growth in some branches resulting from dichotomy. Thus far, no candidate genes have been identified that can be responsible for such a transition from a three-dimensional to a two-dimensional growth pattern.

However, an alternative scenario has been postulated where leaves result not from flattening of dichotomising telomes but from the sterilisation of sporangia [2,8,35]. Indeed, three types of transcription factors, class III HD-ZIP, KANADI, and YABBY, that are known to regulate leaf development, are formed in both leaf and sporangia primordia of lycophytes [8,32,36]; class III HD-ZIP TFs are also formed in the primordia of leaves and sporangia of ferns [8]. These data suggest that during evolution, leaves did not originate from the “planation” of telomes, but from displacement of sporangia from a terminal to a lateral position, placing their origin in a group of founder cells at the periphery of the SAM instead of in the entire SAM. This would have been followed by the sterilisation of some of the lateral sporangia. Based on the expression data described above, it is likely that co-option and/or functional modification of class III HD-ZIP and YABBY TFs in different lineages of early land plants could have played a major role in this modification of a sporangia developmental programme into a leaf developmental programme. The fact that in seedless plants and gymnosperms, *YABBY* homologues are expressed in vegetative and generative organs—in leaf and sporangia primordia of the lycophyte *Huperzia selago* [36], both in ovule integument and bracts of the gymnosperm *Ephedra distachya* [21]—combined with expression of “vegetative” *YABBY*s in both vegetative and reproductive organs of the basal angiosperms *Cabomba caroliniana* and *Amborella trichopoda* [43,49,72], enables to hypothesise that one of YABBY’s putative ancestral functions could have been the regulation of meiosis.

Consequently, it can be hypothesised that the single *YABBY* homologue of early land plants could regulate the development of both leaf and sporangia, and that subsequent duplication and subfunctionalisation led to the emergence of “vegetative” and “reproductive” *YABBY*s in angiosperms. While some authors suggest that only in seed plants, an ancestral shoot-specific network is transformed into a leaf-specific one by YABBY TFs [21,35,55], involvement of YABBY TFs in the regulation of the development of both vegetative and reproductive organs in lycophytes as well as in gymnosperms can be regarded to support the hypothesis of Vasco and co-authors [8] that leaves in different plant lineages could result from sterilization of sporangia since both leaves and sporangia share deep homology. It can also be hypothesised that YABBY TFs which in a lycophyte are expressed in the PZ of the SAM and in the primordia of lateral organs could have been responsible for the functional partitioning of the simplex SAM of early divergent land plants into the CZ with the pool of apical initials and the organogenic PZ. This functional specialisation would have only been possible in the multicellular simplex SAMs. More studies on lycophytes with simplex SAM are required to test this hypothesis.

## Data Availability

Not applicable.
